# Improving the entrepreneurial ability of rural migrant workers returning home in China: Study based on 5,675 questionnaires

**DOI:** 10.1057/s41599-023-01663-5

**Published:** 2023-04-08

**Authors:** Yan Lu, Yuqi Zhou, Pengling Liu

**Affiliations:** 1 College of Economics and Management, Anhui Agricultural University, Hefei, China; 2 The Centre for Research on Science Technology and Education of Agriculture, Anhui Agricultural University, Hefei, China

**Keywords:** Development studies, Economics, Sociology

## Abstract

Innovation and entrepreneurship play a key role in the development of a country’s economy and society. In recent years, along with the comprehensive implementation of China’s rural revitalization strategy, the government has issued many policies to support and benefit farmers, and the investment environment in villages has been greatly improved, attracting more and more migrant workers to return to their hometowns to start businesses. Improving the entrepreneurial ability of China’s returning migrant workers is very important. A review of the previous literature showed that both internal personal factors and external environmental factors can affect the entrepreneurial ability of entrepreneurs. Combining the results of the questionnaire survey, this paper incorporates eight internal personal factors and eight external environmental factors into the index system for assessing entrepreneurial ability and uses a combination of SWOT analysis and AHP analysis to prioritize the factors influencing the entrepreneurial ability of returning migrant workers in China. The results are as follows: (1) The factors influencing the entrepreneurial ability of returnee entrepreneurs could be divided into two types: the individual factors of returnee entrepreneurs, and the external environmental factors. (2) Among the internal factors, technical experience and business management ability are the two main factors influencing entrepreneurial activity. (3) Among the external environmental factors, the opportunity for industrial structure adjustment and low technical content would have a higher influence on the entrepreneurial activity of returnee entrepreneurs. Accordingly, this study proposes some policy recommendations on how to improve the entrepreneurial ability of returnee entrepreneurs. Finally, the research methodology of this paper can be used as a reference for other scholars, and the research results can also be used for administrators to develop relevant policies to better improve the entrepreneurial ability of returnee entrepreneurs.

## Introduction

In the mid-1980s, the first farmers in China were forced to leave their hometowns and go to the city to find work to earn more money to support their families. With the motivation of the first migrant workers, more and more farmers in China went to the city to find work. In 1989, the number of migrant workers in China exceeded 30 million (data source: China National Bureau of Statistics). The phrase, “flood of migrant workers” appeared in the media. Since then, huge numbers of migrant workers have flooded into the townships every year in search of work, and in 2019 there were more than 300 million migrant workers in the townships for all kinds of work (data source: China National Bureau of Statistics). These migrant workers have made great contributions to urban construction and social and economic development. However, since 2020, there has been a “counter-current” phenomenon for migrant workers in China, with more and more migrant workers choosing to return to their hometowns for local employment and entrepreneurship.

First, the main reason for the large number of migrant workers returning to their hometowns is the influence of the epidemic and the economic environment. In 2020–2022, due to COVID-19 and the slow economic growth in China, many enterprises in cities were hard to survive, and there was a sharp decline in employment opportunities. The income of migrant workers working in cities has been greatly reduced. At the same time, the higher cost of living in cities would add to their burden. In such a situation, many migrants begin to return to their hometowns and engage in hometown entrepreneurship. Another important reason for the return of Chinese migrant workers is policy support from the Chinese government. To better encourage migrant workers to return home and start businesses, the Chinese central government has issued a number of preferential policies. Under the guidance of these policies, the number of migrant workers returning home to China has continued to increase. According to data from the National Bureau of Statistics of China, more than 30 million migrant workers returned home in 2020, and a total of 11.2 million people returning to the countryside in 2022, an increase of 43.59% compared to 2018. After returning to their hometowns, these migrant workers generally choose to start businesses and find jobs in the local area. Although the number of migrant workers returning home to start their own businesses is increasing, their success rate is low. According to the research team’s survey data, the 3-year survival rate of migrant workers returning home to start businesses is only 43.49%, and the 5-year survival rate is only 22.71%. In the coming years, the trend of migrant workers returning home to start businesses in China will continue, and the issue of migrant workers returning home to start businesses will become more important.

The existing literature suggests that the entrepreneurial ability of entrepreneurs will directly determine the success of their entrepreneurial activities (Aidin et al., 2022; Shabbir et al., 2016; LY et al., 2014). First, the entrepreneur’s own qualities are the source of entrepreneurial ability and these include, among others, personal creativity (Gibb, 1993), information-gathering skills (Aidin et al., 2022), interpersonal relationships, experiential knowledge (LY et al. 2014), and risk attitude (McClelland, 1965). Second, entrepreneurial capabilities are similarly influenced by external environmental factors (Dabi et al., 2020; Mazzarol et al., 1999), such as cultural background, natural conditions, government policies, and so on. These external environmental factors have a specific impact first on the entrepreneur and then on the entrepreneurial activity. Researchers in China have argued that the low cultural quality, insufficient technical skills and lack of management experience of Chinese returning migrant workers are the main reasons for their low success rate in entrepreneurial activities (LY et al., 2014; Wang and Sun, 2018). Although existing studies have identified a number of factors that influence entrepreneurship, they do not provide a relatively reliable method for assessing the extent to which each of these influences affects entrepreneurship. Therefore, the aim of this paper is to discover a feasible approach that can incorporate both internal individual factors and external environmental factors that affect entrepreneurs’ entrepreneurial capabilities into the research framework, to determine exactly which factors have an impact on entrepreneurs’ innovation capabilities and to what extent these factors affect innovation capabilities, in order to seek the best way to enhance entrepreneurs’ innovation capabilities.

## Literature review

Many scholars believe that successful entrepreneurial activity could effectively promote the development of society and the economy (Aidin et al., 2022; Hosseini et al., 2020). The entrepreneurial ability of entrepreneurs would largely determine the success rate of entrepreneurial activity. The more entrepreneurial skills they have, the stronger their entrepreneurial ability would have and the higher the success rate of entrepreneurship (Soltanifar et al., 2020). The current mainstream view is that a successful entrepreneur should have the following entrepreneurial abilities: creativity (Gibb, 1993), risk-taking (McClelland, 1965), visionary (Kao, 1989), opportunity seeking (Peterson, 1985), and so on. In addition, Nieuwenhuizen believes that entrepreneurial ability is the one for entrepreneurs to create new valuable things with the returns of entrepreneurship through the use of time, capital, experience, psychology, and social risk (Nieuwenhuizen, 2009). Shabbir believes that entrepreneurial ability should have self-esteem, courage, persistence, a desire for immediate results, and the ability to recognize opportunities (Shabbir et al., 2016). Aidin believes that entrepreneurial skills are formed as a result of learning and acquiring information (Aidin et al., 2022). According to Dana, the entrepreneurial ability of entrepreneurs could be cultivated through specific education, so adding entrepreneurship education is good for improving entrepreneurial ability (Dana et al., 2021). Salamzadeh believes that a successful entrepreneur should have the following characteristics: open-mindedness, need for achievement, pragmatism, tolerance of ambiguity, visionary, challenge taking, risk-taking, internal locus of control (Salamzadeh et al., 2014). Guru developed a general model of immigrant entrepreneurial ability that identifies four different entrepreneurial pathways, each characterized by a specific combination of personal resources, network embeddedness, individual habitus, and targeted opportunities (Guru et al., 2020). Some scholars even believe that language skills, relevant experience, resource conditions, and social integration would influence the entrepreneurship of returnee entrepreneurs (Constant et al., 2009; Millar and Choi, 2008).

Other scholars believe that the entrepreneurial ability of entrepreneurs would be affected by external environmental factors in addition to individual internal factors. Dabi believes that the entrepreneurial activity of returnee entrepreneurs would be influenced to some extent by different countries and regions. In other words, the same entrepreneur would have a completely different result for conducting entrepreneurship activity in different countries or regions (Dabi et al., 2020). Through a large number of case studies, Hamilton finds that the entrepreneurial ability of returnee entrepreneurs would be affected by different cultural backgrounds (Hamilton et al., 2008). Mazzarol believes that it would be more comprehensive to study the entrepreneurial ability of entrepreneurs from two aspects: (1) personal characteristics or traits of entrepreneurs; (2) the influence of external factors such as culture, politics, society and economy (Mazzarol et al., 1999). Some scholars believe that the government should provide such an external environment, such as entrepreneurship training and policy support for entrepreneurs (Ashourizadeh et al., 2022).

Based on the study of the above scholars, this paper argues that the entrepreneurial ability of entrepreneurs is composed of individual internal factors and external environmental factors. The individual internal factors determine the strengths and weaknesses of entrepreneurs, while the external environmental factors determine the opportunities and threats faced by entrepreneurs. Entrepreneurial ability depends on the extent to which entrepreneurs can exploit their advantages, seize opportunities and avoid risks. Entrepreneurial ability is the ability of entrepreneurs to conduct certain businesses by allocating all kinds of resources (including the capital, human resources, land, time and social relationship, etc.) in a unique external environment to achieve certain expected entrepreneurial goals. The entrepreneurial ability of entrepreneurs is jointly determined by the personal characteristics (individual internal factors) of entrepreneurs and external environmental factors. Therefore, the article would divide the factors influencing the entrepreneurial ability of returnee entrepreneurs in China into two types: individual internal factors and external environmental factors. Individual internal factors include: (1) capital; (2) education; (3) skills and experience; (4) interpersonal connections; (5) management ability; (6) hard-working spirit; (7) access to information; and (8) communication skills. The external environmental factors include: (1) preferential policy; (2) market risk; (3) government support; (4) external financing conditions; (5) industrial restructuring; (6) industry system; (7) industrial technology content; and (8) market competition.

The manuscript is structured as follows. First, in the introduction, the research background is introduced, and the key issue is raised. Secondly, in the literature review, research views of relevant scholars are introduced, and based on them, the conceptual framework is extracted. Thirdly, by the exploitation of an applied research method, data analysis is done. Finally, the article makes recommendations and draws conclusions.

## Methods

### SWOT and AHP

There are several methods that can be used to analyze and evaluate the key factors that influence the entrepreneurial capacity of migrant workers. The most common method is the Delphi method proposed by T. Gordon and O. Helmer (Gordon and Olaf, 1964), which mainly consists of anonymously collecting the opinions of relevant experts on a given topic. Each expert expresses his or her own opinion under the premise of not communicating with each other, and then the organizer collects, classifies, and counts these opinions, feeds the results back to the experts, and collects their opinions again. This is repeated several times until a more consistent opinion is reached. Tim Padmore and Hervey Gibson (1998), Canadian researchers, used the “Groundings-Enterprises-Markets” (GEM) model to analyze the factors affecting the competitiveness of industrial clusters. In applying the model, they introduced a spider-web diagram to identify six factors (i.e. the six corners of the spider-web model) and assigned a value (ranging from 1 to 10 points) to each factor. The ‘factor pair’ was then calculated to determine the score of the GEM model, and on this basis, the impact of the six factors on industrial clusters is determined. The advantages of this scoring method are that it is easy to use and the scoring results are very intuitive (spider graph), while the disadvantages are that there is a high degree of subjectivity in the scoring of each influencing factor, which to some extent affects the accuracy of the scoring results. In addition, the logit model proposed by Fitzpatrick (Fitzpatrick, 1932) and the probit model proposed by Eichengreen (Eichengreen et al., 1996) can also be used to analyze the degree of impact of influencing factors. Yang Jinxiu (Yang et al., 2010) and others used the Logit model to analyze the factors affecting migrant workers in China. Xu Tianzeng (Xu, 2010) used the probit model to analyze the factors influencing the willingness of migrant workers to settle in Beijing. According to the literature, logit and probit models are actually used to analyze binary discrete phenomena. Generally, the positive (or qualified) qualitative index is assigned to 1, and the negative index is assigned to 0. Then, the regression results of Logit or Probit are obtained according to the statistical data, and then the adjoint probability value of the influencing factors in the results is used to judge which factors have a higher level of influence. However, these two models also have their own shortcomings: When using these two models for multi-factor analysis, it may cause information distortion of multiple discrete variables, and also cause endogenous problems of the probit model, which are difficult to improve by other methods. Professor Saaty (Saaty, 1977; Saaty, 1980), an American operations researcher, suggested the importance of using the analytic hierarchy process (AHP) to analyze the influencing factors. This is a combination of qualitative and quantitative methods, and its core idea is to calculate the combination weight of the constituent elements of each level to the overall goal through five steps: clarifying the problem, establishing the hierarchical analysis structure model, constructing the judgment matrix, performing hierarchical single ranking and hierarchical total ranking, and the combination weight of each element directly reflects the degree of its impact. As the influence elements in the hierarchical analysis process are generally determined by Delphi or other qualitative methods, the determination of the influence elements is highly subjective and may have a certain impact on the results.

In order to make the analysis as objective as possible, this paper will use a combination of two methods, SWOT analysis and AHP analysis, to find out the key factors affecting the entrepreneurial ability of returning migrant workers. The specific operation process is as follows. First, the SWOT analysis method is used to analyze the key factors influencing the entrepreneurial ability of returning migrant workers based on the data from the questionnaire survey. Then, the AHP analysis method is used to analyze the factors affecting the entrepreneurial ability of migrant workers, identify the key factors and propose relevant countermeasures.

### Data collection

From July 2018 to October 2022, the research team conducted a questionnaire survey on the entrepreneurial ability of migrant workers returning home. The research team surveyed four provinces in China with large numbers of migrant workers, namely Sichuan, Henan, Jiangxi, and Anhui. The research team was divided into four groups and conducted 56 consecutive surveys. The questionnaire survey covered 36 prefecture-level cities in the four provinces. A total of 6720 questionnaires were distributed, and 5675 valid questionnaires were actually collected and sorted. The effective rate of the questionnaire was 84.45%, including 3405 successful entrepreneurs and 2270 entrepreneurial losers.

The survey data are shown in Table 1, the average age of migrant workers returning home to start businesses is about 43 years old, with an average of 2.5 workers per household (average data) and an average population of about 5 people per household. Of the survey sample, 57.62% had a junior high school education or less, 27.67% had a senior high school education, and 14.71% had a college education or more. At the same time, 74.78% of the migrant workers who returned home to start businesses were educated and 75.38% had skills.

**Table 1:** Characteristics of migrant workers returning home for business start-ups.

Entrepreneur’s quality			Successful entrepreneurs	Entrepreneurial losers
Endowment	Household labor force	Age of entrepreneur (years)	42.17	43.35
		Labor force (person/household)	2.56	2.41
		Total population (person/household)	4.74	4.45
	Education composition (%)	Illiteracy	0	16.73
		Primary school	15.01	42.01
		Junior high school	19.75	33.15
		High school	42.24	5.82
		University	23.01	2.29
Technical conditions	Trained (%)		90.27	51.72
	Skilled (%)		93.76	47.79

Source: Calculated according to the data obtained from the questionnaire survey (2018–2022).

### Data analysis procedures

As mentioned above, this paper first uses SWOT analysis to identify the strengths, weaknesses, opportunities, and threats of entrepreneurship among returning migrant workers in China and then uses AHP analysis to rank the importance of these factors. The specific procedure is as follows.

#### SWOT analysis

The questionnaire included 16 internal factors (A1–A8) and external factors (B1–B8) that affect the entrepreneurial ability of migrant workers returning home (LY et al., 2014). During the survey, the sample staff decided whether to select “significant impact” according to their own judgment. According to the descending order of the number of people who selected “significant impact”, the internal factors and external factors were sorted, respectively, to form Table 2.

**Table 2:** Survey and statistics on the importance of factors affecting the entrepreneurial ability of returning migrant workers.

S/N	Entrepreneurial factors	Number of people who choose “significant impact”	Proportion (%)	Self-evaluation level
A1	Capital	5235	92.24669604	−
A2	Education	5167	91.04845815	−
A3	Skills and experience	4843	85.33920705	+
A4	Interpersonal connections	4533	79.87665198	+
A5	Management ability	4476	78.8722467	−
A6	Hard-working spirit	4366	76.9339207	+
A7	Access to information	2421	42.66079295	+
A8	Communication skills	2165	38.14977974	−
B1	Preferential policy	5342	94.13215859	+
B2	Market risk	5087	89.63876652	−
B3	Government support	5046	88.91629956	+
B4	External financing conditions	4653	81.99118943	−
B5	Industrial restructuring	4628	81.55066079	+
B6	Industry system	4368	76.969163	+
B7	Industrial technology content	4298	75.73568282	−
B8	Market competition	3207	56.51101322	+

Source: Calculated according to the data obtained from the questionnaire survey (2018–2022).

To find out the key factors of migrant workers returning home to start businesses, these 16 factors need to be classified and screened, and the SWOT model is a better choice (LY et al., 2014). In Table 2, the entrepreneurial factors of No. A1–A8 are the quality of returning entrepreneurs, which determines the strengths and weaknesses of returning entrepreneurs (Wang and Sun, 2018). The “self-evaluation level” is the subjective evaluation of the survey sample personnel on their own factors. If the “self-evaluation level” is assigned “+“, it indicates that the survey sample personnel believe that they have a relative strength in this factor; if the value is “−“, it means that the survey sample personnel believe that they have a relative weakness in this factor. Entrepreneurial factors of No. B1–B8 are the external environmental factors of entrepreneurial ability, which determine the opportunities or threats in the entrepreneurial environment. “Self-assessment level” is the subjective assessment of the environmental factors by the sample personnel. If the sample persons believe that this factor is favorable for their entrepreneurial ability, it is assigned a value of ‘+‘ as an opportunity factor; if the factor is unfavorable for the entrepreneurial ability, it is assigned a value of ‘−‘ as a threat factor.

According to Table 2, from the entrepreneurial factors A1–A8, three entrepreneurial factors with the highest proportion of “total self-assessment level” assigned as “+“ are selected as “strength factors” and three entrepreneurial factors with the highest proportion of “total self-assessment level” assigned as “−“ are selected as “weakness factors”; from entrepreneurial factors B1–B8, three entrepreneurial factors with the highest proportion of “overall self-assessment level” assigned as “+“ were selected as “opportunity factors”, and three entrepreneurial factors with the highest proportion of “overall self-assessment level” assigned as “−“ were selected as “threat factors”. After screening and sorting, the SWOT matrix of migrant workers returning home to start a business was obtained (see Table 3).

**Table 3:** SWOT matrix of migrant workers returning home to start businesses.

Strengths	Weaknesses
Skills and experience (S1)	Insufficient fund (W1)
Interpersonal connections (S2)	Low cultural quality (W2)
Hard-working spirit (S3)	Low management ability (W3)
**Opportunities**	**Threats**
Government incentives (O1)	Market risk (T1)
Government support (O2)	External financing difficulties (T2)
Industrial restructuring (O3)	Low technology content (T3)

##### Strength factors

Compared to other farmers, the advantages of migrant workers returning home to start businesses are mainly manifested in the strengths of skills and experience (S1), interpersonal connections (S2), and hard-working spirit (S3) as entrepreneurial strengths. First of all, for migrant workers returning home to start businesses, having certain skills and experience is the necessary basis for entrepreneurship. In the survey, almost all entrepreneurial farmers have received professional skills training and have been engaged in related professional work for more than one year. Their skills involve handicrafts, catering services, planting, aquaculture, deep processing of agricultural products, sales of building materials, machinery and vehicle maintenance, and other fields, which lay a good foundation for entrepreneurship. Secondly, migrant workers returning home to start businesses need to have good interpersonal connections (Obi-Anike et al., 2022). Because of geopolitical and blood ties, migrant workers who work together in cities are more likely to establish close cooperative relationships, which is why the proportion of them who return home together to start businesses is very high, almost 90% (the data from the Ministry of Agriculture and Rural Affairs of the People’s Republic of China). In addition, most of these people have good local connections, according to interviews with local people. “Interpersonal connections are helpful for doing things” is well reflected in the process of entrepreneurship: It is easier to get help, because there are many interpersonal connections, and the relevant licensing procedures are relatively smooth. Finally, migrant workers who return home to start businesses have a strong spirit of hard work. The main result of the survey is that in the process of entrepreneurship, all things are done by migrant workers themselves, they always work overtime, even if they have money, they still save money, maximize cost savings, and accumulate wealth through diligence and saving.

##### Weakness factors

The weaknesses of returning entrepreneurs are mainly manifested in insufficient funds (W1), low cultural quality (W2), and low management ability (W3). (1) Although migrant workers accumulate certain funds in the stage of going out to work, and their income is higher than that of local farmers, these funds are far from enough for entrepreneurship. The survey found that most entrepreneurship projects require about 200,000 yuan (RMB) or even more, so the lack of funds is a major problem for migrant workers returning home to start businesses. (2) The cultural quality of migrant workers returning home is generally not high. Survey data shows that 57.62% of them have less than primary and junior high school education. Low cultural quality makes entrepreneurs have a weak ability to learn skills, low ability to gather information, difficulty to find and grasp market opportunities in time, and difficulty to make accurate judgments and formulate correct strategies in a changeable competitive environment. (3) The enterprises set up by migrant workers returning home are usually traditional workshop enterprises and relatives and friends participate in the operation and management. Such enterprises have small business scale, weak ability to resist risks, and unreasonable management mechanisms, which will eventually lead to difficulties in the development and growth of enterprises. In the investigation of enterprises founded by migrant workers returning home, without exception, relatives, and friends are arranged in important management positions: parents engage in supervision, wives engage in finance, friends engage in public relations, and so on. In addition, migrant workers returning home still have the business concept of small farmers' awareness, short-sighted vision, and weak market awareness.

##### Opportunity factors

The entrepreneurial opportunities faced by migrant workers returning home are mainly manifested in preferential policies (O1), government support (O2), and industrial restructuring (O3). The main preferential policies are as follows: In January 2018, the State Council issued the “Opinions on Further Supporting Migrant Workers and Other People Returning to the Rural Area to Start Their Own Business”, which supports farmers to start businesses from the implementation of preferential policies, infrastructure improvement, technical training, rural financial system support, and other aspects. On June 17, 2020, nine departments such as the Ministry of Agriculture and Rural Affairs and the National Development and Reform Commission jointly issued the “Opinions on Deepening the Training of Rural Innovation and Entrepreneurship Leaders”, which clearly stated that support policies should focus on “people, land and money”. It is expected that by 2025, more than 15 million entrepreneurs will return home and create about 60 million jobs. On November 16, 2022, the Ministry of Human Resources and Social Security, the National Development and Reform Commission, the Ministry of Finance, the Ministry of Agriculture and Rural Affairs and the National Rural Revitalization Administration issued the “Implementation Opinions on Further Supporting Migrant Workers’ Employment and Entrepreneurship”, taking various measures to stabilize growth and employment, and further support the employment and entrepreneurship of migrant workers and people out of poverty.

The main incentive measures are as follows: in 2018, the No. 1 document of the Central Committee “Opinions on Implementing the Rural Revitalization Strategy” proposed that “Implementing Rural Employment and Entrepreneurship” is an important way to promote the transfer of rural labor and increase farmers’ income, and encourage all kinds of talents to innovate and start businesses in rural areas. In 2019, the No. 1 document of the Central Committee “Several Opinions on Implementing the Rural Revitalization Strategy”. document of the Central Committee “Several Opinions on Giving Priority to the Development of Agriculture and Rural Areas and Doing Good Work in Agriculture, Rural Areas and Farmers” pointed out that migrant workers and other talents should be encouraged to return to the countryside for innovation and entrepreneurship. Under the leadership of the Party Central Committee, local governments have not only introduced many policies to encourage migrant workers to return home and start businesses but also strengthened rural infrastructure construction, creating a good external environment for migrant workers to return home and start businesses. In 2020, the No.1 document of the Central Committee proposed to “thoroughly implement the training of rural innovation and entrepreneurship leaders, and include eligible migrant workers returning home in the scope of one-time entrepreneurship subsidies”. The No.1 document of the Central Committee in 2021 proposed to “attract talents from all aspects of cities to rural areas for entrepreneurship and innovation, participate in the rural revitalization and modern agricultural construction, and encourage local governments to build entrepreneurship parks and incubation and training bases”. In 2022, the No.1 document of the Central Committee proposes to “promote the employment and entrepreneurship of farmers locally and nearby, promote the construction of entrepreneurship parks for returning home and entering the countryside, and implement various support policies”.

##### Threat factors

Threat factors are manifested in various adverse factors of the external environment on entrepreneurship, mainly in three aspects. (1) Market risk is high. At present, the market environment is complex and changeable. If we choose some agricultural entrepreneurship projects, we will also be affected by natural disasters, and the risk for migrant workers to start businesses will increase significantly. (2) External financing is difficult. As farmers have less property and lack long-term stable sources of income, it is also difficult for migrant workers to obtain loans from the financial sector for entrepreneurship. With the support of national policies, individual banks allow entrepreneurial farmers to have a certain amount of credit loans, but the amount is small, which cannot solve practical problems. Although some micro-finance companies can provide large loans, the interest rate is too high and the repayment period is relatively short, which cannot solve the problem but increases the economic burden and risk of entrepreneurs. (3) The technical level of the agricultural industry is low. Due to historical and other reasons, the overall level of agricultural development in China is low, which leads to the low technical level of entrepreneurial projects chosen by migrant workers returning home, which is difficult to compete with other competitive enterprises. Therefore, market risk (T1), difficulty in external financing (T2), and low technology content (T3) are identified as the main threats to migrant workers to start businesses.

#### AHP analysis

Analytic hierarchy process (AHP) is a proposed hierarchical weighted decision analysis method proposed. Its advantage is that it combines qualitative and quantitative analysis well, and provides quantifiable analysis results for complex and fuzzy decision-making problems. The Analytic Hierarchy Process ranks the above 12 key factors (SWOT factors), comprehensively evaluates the 12 key factors, measures the priority of these factors by the same standard, and finally determines the priority order. The steps are as follows.

##### Set up a hierarchical model

**Fig. 1:**
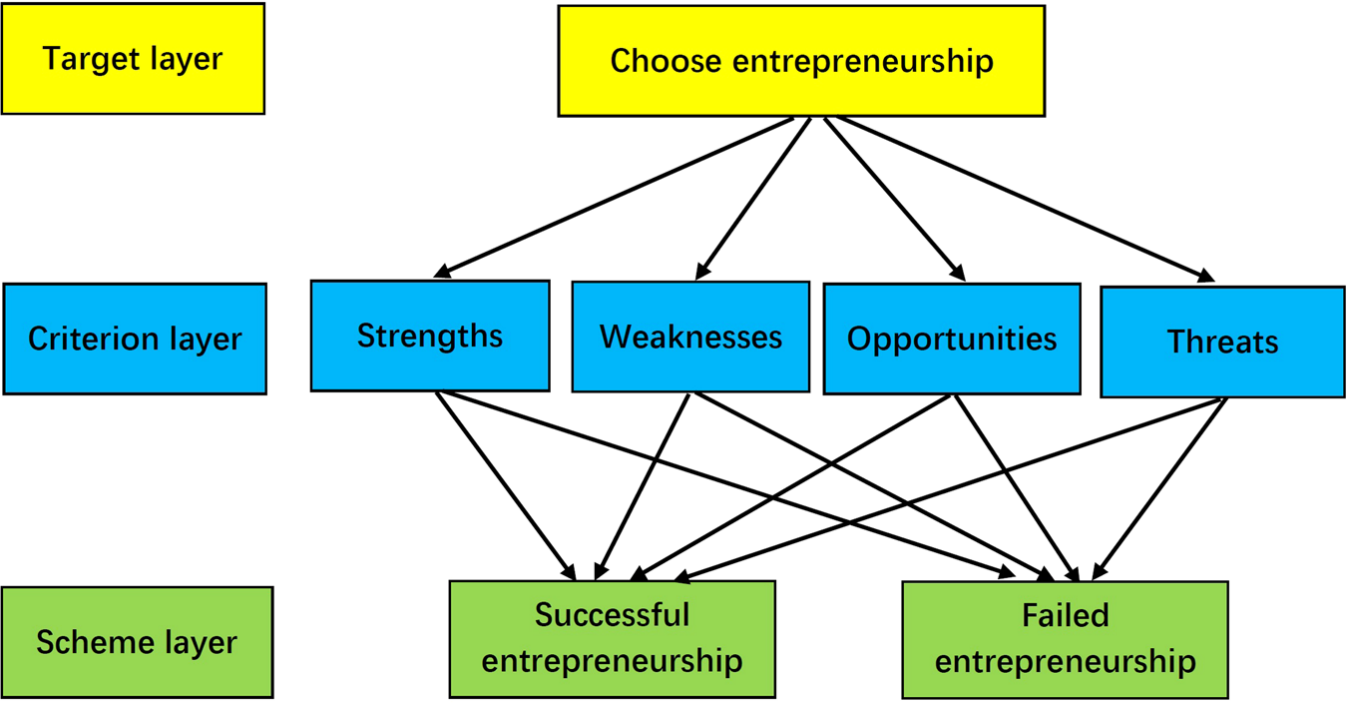
Hierarchical structure of entrepreneurial ability of returning migrant workers. The target layer is the key problem to be solved by the decision, which is the choice of returning migrant workers to start business; the criterion layer is the main factors influencing the migrant workers’ entrepreneurship (SWOT elements); and the scheme layer is the development of suitable alternatives, which will directly determine the success or failure of the entrepreneurship.

The structure of the model is shown in Fig. 1, and the top layer is the key problem to be solved by the model: the results of migrant workers’ choice of entrepreneurship; the middle layer is four factors affecting migrant workers’ entrepreneurial ability (including 12 SWOT factors); the basic layer, also known as the scheme layer, is the result of the choice: success or failure.

##### Selection of key factors for entrepreneurial ability

The strengths, weaknesses, opportunities, and threats of migrant workers returning home to start businesses are analyzed by using the SWOT tool, and the key factors included in these four groups are listed. The key strengths in determining the entrepreneurial ability of migrant workers returning home include skills and experience (S1), interpersonal connections (S2), and hard-working spirit (S3), the key weaknesses include insufficient funds (W1), low cultural quality (W2) and low hard-working spirit and management ability (W3), the key opportunity factors include preferential policies (O1), encouragement (O2) and industrial restructuring (O3) from the government, and the key threat factors include market risk (T1), difficulty in external financing (T2) and low technology content (T3).

##### The computational logic of priority

In the strength group, the weakness group, the opportunity group, and the threats group, the three key factors are combined to compare the priority, the operation matrix of the internal elements of each group is established, and then the priority weights of these factors are calculated. When the priority of pairwise combination of key factors is calculated, there may be inconsistency in the comparison results, which requires determining the elastic range of inconsistency (LY et al., 2014). Generally, the consistency index CI of the judgment matrix is calculated to test whether it has satisfactory consistency results, and its calculation formula is CI = (*λ*_max−*n*_)/(*n*−1). When CI = 0, there is complete consistency; when CI is close to 0, there is satisfactory consistency; the greater the CI, the more significant the inconsistency. Since CI is related to *n*, the consistency ratio CR = CI/RI is used to measure whether the judgment matrices of different orders have satisfactory consistency. It should be added here that RI is a correction value or degree of freedom. Due to many factors, the dimension of pairwise comparison will be relatively large, and the consistency of CI judgment will be worse. Therefore, the requirement of consistency of the comparison matrix can be moderately reduced, so the correction value RI must be introduced. When CR is used to judge the consistency of the matrix, only when CR is <0.1, the matrix has satisfactory consistency and can pass the consistency test.

Finally, the priority weights of these factors are calculated using the characteristic value method and the four SWOT groups are compared in pairs for an overall hierarchical ranking.

## Results

### Intra-group ranking of SWOT factors

The intra-group ranking of the key factors depends on the value of the judgment matrix. As the first step in the AHP analysis, the judgment matrix of the SWOT elements of the entrepreneurial capacity of returning migrant workers in China is shown in Table 4 (including 4 tables).

**Table 4:** Judgment matrix.

Strength group			
Strength group	S1	S2	S3
S1	1	2	1/4
S2	1/2	1	4
S3	1/4	1/4	1

Weakness group			
Weakness group	W1	W2	W3
W1	1	1/3	1/5
W2	3	1	1/3
W3	5	3	1

Opportunity group			
Opportunity group	O1	O2	O3
O1	1	4	1/3
O2	1/4	1	1/8
O3	3	8	1

Threat group			
Threat group	T1	T2	T3
T1	1	1	1/3
T2	1	1	1/5
T3	3	5	1

Table 4 shows the relative importance values of the factors in each group, i.e. the ratio of the relative importance of each element within the group. For example, in Table 4, the relative importance value of S1 to S2 is 2, indicating that factor S1 is more important than factor S2 (S1 > S2); the relative importance value of S2 to S3 is 4, indicating that factor S2 is more important than factor S3 (S2 > S3), from which it can be deduced that the importance ranking of strength group is S1 > S2 > S3. Similarly, the importance ranking of the weakness group is W3 > W2 > W1, the importance ranking of the opportunity group is O3 > O1 > O2, and the importance ranking of the threat group is T3 > T1 = T2.

### Cross-group ranking of SWOT factors

The cross-group ranking of the SWOT factors is shown in Table 5.

**Table 5:** Priority results of key factors for entrepreneurial ability of returning migrant workers.

SWOT group	Group priorities	Key factors	CR	Prioritization of factors within each group	Overall priority of factors
Strengths	0.2868	Skill experience S1	0.0164	0.5821	0.1724
		Interpersonal connections S2		0.3287	0.0887
		Hard-working spirit S3		0.1113	0.0307
Weaknesses	0.2376	Insufficient fund W1	0.0187	0.1103	0.0236
		Low literacy W2		0.2613	0.0714
		Low management ability W3		0.6241	0.1511
Opportunities	0.2553	Government incentives O1	0.0154	0.2489	0.0573
		Government support O2		0.0762	0.0186
		Industrial restructuring O3		0.6682	0.1762
Threats	0.2455	Market risks T1	0.0136	0.1764	0.0532
		External financing difficulties T2		0.1644	0.0369
		Low technology contents T3		0.7458	0.1750

As shown in Table 5, the CR value of each factor in the evaluation matrix is <0.1, so the matrix has a good consistency, and the analysis results are acceptable. According to the priority results of four types of key factors in each group, skill experience in the strength group (S1), low management ability in the weakness group (W3), industrial restructuring in the opportunity group (O3) and low technology content in the threat group (T3) have the most significant impact on entrepreneurial ability. According to the comprehensive overall priority results of 12 factors, the priority of each factor (the importance of the impact on entrepreneurial ability) is as follows O3 > T3 > S1 > W3 > S2 > W2 > O1 > T1 > T2 > S3 > W1 > O2. It is worth mentioning here that the results of intra-group priority ranking of threat groups show that T1 and T2 have the same priority (T1 = T2), but in the inter-group priority ranking results, T1 is better than T2 (T1 > T2), which indicates that T1 and T2 have the same influence on the entrepreneurial ability of Chinese returning migrant workers without considering the interference of other factors, and when the influence of multiple factors is considered, T1 will have a more significant influence on the entrepreneurial ability of Chinese returning migrant workers compared to T2. In reality, it is clear that entrepreneurial ability is influenced by multiple factors, and therefore T1 has a higher priority than T2.

Overall, the opportunity of industrial restructuring, the low technology content of the industry, skills and experience, and management ability are the key factors determining the entrepreneurial ability success or failure of returning migrant workers.

## Discussion and conclusions

### Discussion

#### Implications for theory

This paper divides the factors affecting the entrepreneurial ability of returning migrant workers into two main categories: individual internal factors and external environmental factors. Among these, the individual internal factors of entrepreneurial ability of returning migrant workers include: (1) capital; (2) education; (3) skills and experience; (4) interpersonal connections; (5) management ability; (6) hard-working spirit; (7) access to information; (8) communication skills. This is in line with Gibb (1993), Shabbir et al. (2016), Nieuwenhuizen (2009), and Aidin et al. (2022). And the external environmental factors of entrepreneurial ability of returning migrant workers include: (1) preferential policy; (2) market risk; (3) government support; (4) external financing conditions; (5) industrial restructuring; (6) industry system; (7) industrial technology content; and (8) market competition. This is in line with scholars such as Dabi et al. (2020), Hamilton et al. (2008), Kloosterman et al. (1998), Mazzarol et al. (1999), and Ashourizadeh et al. (2022). Although the indicator system established in this paper is consistent with the current research findings of mainstream scholars, they only focus on a few key factors, which tends to create a “blind spot” in the research perspective. Based on the results of the questionnaire survey, this paper innovatively adds more factors to the indicator system to make it more reasonable.

Second, this study is the first attempt to use the combination of SWOT–AHP methods to study entrepreneurial ability issues. Scholars such as Helms et al. (2011), Karami and Agahi (2018), and Noel et al. (2021) use SWOT analysis for entrepreneurial ability, and Zeynep (2018) and Krakowiak-Bal et al. (2017) use AHP analysis to study entrepreneurial ability issues. Only a few scholars, such as Stefan et al. (2021) and others, have used SWOT–AHP analysis to analyze this issue, but these studies focus more on the impact of the entrepreneurial environment on entrepreneurial ability, which affects the results to some extent. The innovation of this paper is that the internal personal factors and external environmental factors influencing entrepreneurial ability are equally and uniformly included in the same analytical framework. The SWOT–AHP analysis is used to prioritize these influencing factors in order to identify the ‘shortcomings’ of entrepreneurs’ entrepreneurial ability and to make more targeted recommendations for improvement.

Thirdly, taking into account the availability of data and literature, the author has tried to analyze the problems of entrepreneurial ability in America, Britain, Japan, and Korea with the method and finds that the effects are good. Meanwhile, the study finds that the internal factors of entrepreneurs (personal entrepreneurial ability) in America, Britain, and Japan are stronger than those of the Chinese and Korean entrepreneurs; and the external environmental factors of China and America are more conducive to the entrepreneurial activity of entrepreneurs than those in Britain, Japan, and Korea. Therefore, this method is also used for the comparative analysis of the entrepreneurial ability of entrepreneurs in different regions. It should be noted that in studying the problems of entrepreneurial ability with the indicator system of this article, due to a gap in the political, economic, social, and cultural environment in different countries and regions, it will have a better effect if other scholars adapt the indicator system to the research needs according to the environmental characteristics of the research object.

#### Policy recommendations

For migrant workers returning home to start businesses, opportunities and threats coexist, strengths and weaknesses coexist, so seizing opportunities, making full use of their own strengths, compensating for weaknesses, and avoiding threats are the inevitable choices for successful entrepreneurship. Based on the above conclusions, this paper makes the following policy recommendations to improve the entrepreneurial success rate of returning migrant workers.

First, take the supply-side structural reform as an opportunity to promote the adjustment of agricultural industrial structure and create more and better opportunities for entrepreneurship. Although the supply of most agricultural products in China can meet the market demand, the problems of low efficiency of agricultural production, lower quality of agricultural products, the coexistence of excessive and insufficient seasonal supply of some agricultural products, and poor safety of agricultural products are more prominent. Taking the structural adjustment of the supply side of agricultural products as an opportunity, the government has introduced more and better policies to encourage migrant workers returning home to choose appropriate projects when starting businesses, promote the agricultural industrial restructuring, optimize the allocation of rural resources, and improve the efficiency and safety of agricultural product supply. The specific measures are as follows: (1) encourage farmers to develop high-efficiency agricultural projects and vigorously support high-quality and characteristic agricultural product projects; (2) when starting a business, migrant workers should emphasize on improving the quality of agricultural products and have awareness of food safety production; and (3) cultivate migrant workers’ awareness of market competition, timely adjust the production structure of agricultural products according to the market situation, avoid market risks, and improve the success rate of migrant workers returning home.

Second, improve the overall technical level of entrepreneurship and promote the development of characteristic industries. At present, the technological content of business projects chosen by migrant workers returning home is generally low, the problem of project homogenization is prominent, and vicious competition is common. Therefore, when starting a business, farmers must pay attention to improving the technological level of enterprises, developing characteristic industries, and improving their core competitiveness. The government should be market-oriented, formulate a reasonable long-term plan for local entrepreneurship projects, coordinate relations, encourage entrepreneurs to develop characteristic industries, avoid homogenization of entrepreneurship projects, and optimize the allocation of resources. Promote the integration of relevant entrepreneurship projects, which can not only enhance the strength of enterprises and expand the scale of enterprises but also jointly introduce and develop new technologies and spread risks.

Third, migrant workers are the center of entrepreneurial activities, their skill level and management ability are improved through training. Migrant workers are the core of entrepreneurial activities and play a crucial role in entrepreneurial activities. The skills and experience of migrant workers returning home have an important impact on the success rate of entrepreneurship, so improving the entrepreneurial skills and experience of migrant workers through training plays a positive role in entrepreneurial activities. On the one hand, we should strengthen the training of practical skills such as planting, aquaculture, and processing of agricultural products. On the other hand, we should strengthen knowledge training in economic regulations, industry and trade, taxation, finance, science and technology, quality, labor, and other aspects to improve operational and management capabilities. Through such training, entrepreneurs can master more entrepreneurial skills, broaden their horizons, change their ideas, improve their entrepreneurial psychological quality, and enhance their ability to control the market.

Fourth, further, develop the financing channel for returnee entrepreneurs. Although returnee entrepreneurs have extensive human and social resources, people would not borrow money from their acquaintances due to traditional Chinese culture. At the same time, there is an imperfect credit rating and management system construction in China, so the actual financial support available to entrepreneurs is also very limited. Some scholars argue that for returnee entrepreneurs, raising capital through informal economic activities is a feasible and effective approach to solving the lack of venture capital (Kloosterman et al., 1998). Some scholars believe that formal finance is more conducive to innovation than informal sources (Ashourizadeh et al., 2022). In China, it is less costly and less risky to raise capital from formal financing channels (such as banks), but there would be complicated procedures and a long time to acquire capital; while there would be relatively simpler procedures and immediate acquisition of capital in the informal financing channels (such as private loans), but there would be higher cost and greater risk. Therefore, this article argues that the government could take “two measures” to provide some support to returnee entrepreneurs in the financing policy. First, simplify the procedure of the formal financing channels and further accelerate the speed of lending. Second, strengthen the supervision of informal financing channels and standardize the business of informal financing enterprises, so as to control the risk and fully ensure the profit of returnee entrepreneurs.

Fifth, guide the development model of “entrepreneurship with employment” by relying on blood, geography, and other interpersonal connections. Farmers attach great importance to geopolitical and blood ties. They usually work in groups of a few, a few dozen, a few dozen, or even larger, with their fellow villagers or relatives in the same town. They help and care for each other in the city, forming a closer and stronger network of interpersonal ties. Therefore, when migrant workers return home to start a business, it is often a group of people returning home to start a business together, and it can also encourage more local relatives and friends to work in their own businesses. The main feature of this “entrepreneurship drives employment” model is that the relationship between entrepreneurs and employees is relatively close, the degree of mutual trust is relatively high, and the effect of promoting employment is very significant. Thanks to this close network, entrepreneurs are highly motivated, entrepreneurial projects are relatively durable, and their stability is relatively good. However, this model also poses employment and management problems for enterprises. Enterprises need to strengthen the training of managers. If necessary, they can consider hiring competent professional managers for key management positions in order to solve management problems effectively.

In addition, the government should improve rural infrastructure construction and the social security system to create a good software and hardware environment for migrant workers to start businesses. The government should also introduce more policies to promote entrepreneurship, strengthen publicity, create a good entrepreneurial atmosphere, actively guide young college students and a new generation of migrant workers to return home to start businesses, and inject new vitality into returning home to start businesses.

### Conclusions

This paper provides preliminary thoughts on the factors that influence the entrepreneurial abilities of returning migrant workers in China and how to enhance their entrepreneurial capabilities. Existing literature has examined the relationship between entrepreneurial ability and influencing factors from a single perspective, either from the entrepreneur’s own internal factors or from external environmental factors, and reached specific conclusions. However, in reality, entrepreneurs’ entrepreneurial ability is influenced by a combination of their own internal factors and external environmental factors. This paper fills these gaps. Further prioritizing the factors influencing the entrepreneurial ability of returning migrant workers in China, the results show that industrial restructuring, low technology contents, skill experience, and low management ability have a very significant impact on entrepreneurial ability; interpersonal connections, low literacy, government incentives, and market risks have a medium degree of influence on entrepreneurial ability; while, external financing difficulties, hard-working spirit, insufficient fund, and government support have low influence on entrepreneurial ability. Based on the conclusions, this paper proposes specific policy recommendations to improve the entrepreneurial ability of returning migrant workers in China. It is hoped that the indicator system developed, and the research methodology used in this paper will serve as a reference for other scholars conducting related research in order to better advance research in this area. The results of this research can also be used by administrators to develop relevant policies to better improve the entrepreneurial capabilities of entrepreneurs.

## Data Availability

The datasets generated during and/or analyzed during the current study are available from the corresponding author upon reasonable request.
